# Prevalence of Osteoporosis in Elderly Men: Large Real-World Data Addressing the Current Screening Evidence Gap

**DOI:** 10.1007/s11657-026-01701-0

**Published:** 2026-04-13

**Authors:** Vanessa Rouach, Ziv Versano, Meital Sasson, Yona Greenman, Arnon Makori, Gabriel Chodick, Inbal Goldshtein

**Affiliations:** 1 Institute of Endocrinology, Metabolism and Hypertension, Tel Aviv Sourasky Medical Center, Tel Aviv, Israel; 2 The Dr. Miriam and Sheldon G. Adelson School of Medicine, The Gray School), Tel Aviv University, Tel Aviv, Israel; 3 Assuta Medical Centers, Tel Aviv, Israel; 4 School of Public Health, Gray Faculty of Medical & Health Sciences, Tel Aviv University, Tel Aviv, Israel; 5 KI Research Institute, Kfar Malal, Israel; 6 Dina Recanati School of Medicine, Reichman University, Herzliya, Israel

**Keywords:** Osteoporosis, Men, Bone mineral density, DXA screening, Screening recommendations

## Abstract

***Summary*:**

Evidence supporting osteoporosis screening in older men remains limited. In a large real-world cohort of 29,906 men aged ≥ 70 undergoing DXA screening, osteoporosis was identified in 16.5% and osteopenia in 51.3%, with prevalence increasing with age. These findings indicate that a substantial proportion of older men have low bone mass and are therefore at increased risk of fracture.

**Purpose:**

Evidence supporting routine osteoporosis screening in older men remains limited. We aimed to evaluate the prevalence and skeletal distribution of low bone mineral density (BMD) in elderly men within a large healthcare system where an age-based DXA screening recommendation has been implemented.

**Methods:**

We conducted a retrospective cohort study of DXA examinations performed between 2014 and 2024 at Assuta Medical Centers within Maccabi Health Services (MHS). MHS implemented a computerized clinical recommendation within the electronic medical record supporting DXA screening in men aged ≥ 70 years approximately a decade ago. To approximate primary age-based screening conditions, individuals with prior DXA examinations before age 70, oncologic disease, prior fracture, chemotherapy or radiotherapy exposure, and other causes of secondary osteoporosis were excluded. Additional exclusions for inflammatory disease, chronic glucocorticoid therapy, and endocrine disorders were applied using structured diagnostic fields and referral free-text data. Participants were required to have at least two valid skeletal site-groups available for analysis.

**Results:**

Among a total of 29,906 eligible individuals who underwent DXA, osteoporosis prevalence was 15.0% at the femoral neck, 6.1% at the total hip, and 3.8% at the lumbar spine. Using the lowest T-score definition, overall osteoporosis prevalence was 16.5% and 51.3% of participants demonstrated osteopenia. Femoral neck osteoporosis increased from 12.4% in men aged 70–74 years to 26.1% in men aged ≥ 80 years.

**Conclusion:**

In a large real-world cohort approximating systematic screening conditions, nearly one in six older men met diagnostic criteria for osteoporosis. These data help address the current evidence gap regarding osteoporosis screening in men.

## Introduction

Osteoporosis has traditionally been viewed as a disease predominantly affecting women; however, its burden in men is substantial and frequently underrecognized. Approximately one in five osteoporotic fractures worldwide occurs in men (1), and hip fractures in men are associated with higher short-term mortality compared with women (2). Male patients also demonstrate lower rates of osteoporosis diagnosis and treatment following fragility fractures, suggesting persistent gaps in detection and management of skeletal fragility (3, 4). Major professional societies recommend bone mineral density (BMD) testing in older men. The Endocrine Society recommends DXA screening in men aged ≥ 70 years and in younger men with clinical risk factors (5). The International Society for Clinical Densitometry (ISCD) similarly supports BMD testing in men aged ≥ 70 years (6). The Bone Health and Osteoporosis Foundation also recommends fracture risk assessment and BMD evaluation in aging men (7). Despite these recommendations, implementation remains inconsistent, and screening rates in men remain substantially lower than in women across many healthcare systems. Importantly, the 2025 U.S. Preventive Services Task Force (USPSTF) concluded that current evidence is insufficient to assess the balance of benefits and harms of routine osteoporosis screening in men (8). This conclusion reflects limited population-based screening data rather than absence of disease burden. Epidemiologic data from large male cohorts, including the Osteoporotic Fractures in Men (MrOS) study, as well as population-based surveys such as NHANES, have demonstrated that osteoporosis and low bone mass are common in older men, with prevalence varying by age and skeletal site (9–12). However, many prior studies were conducted in mixed-risk populations or relied on opportunistic referral patterns, potentially limiting generalizability to screening contexts.

Understanding the magnitude and skeletal distribution of osteoporosis in older men is essential for informing screening discussions. Israel’s centralized healthcare system provides a unique opportunity to examine low BMD prevalence under quasi-systematic screening conditions. Within Maccabi Health Services, computerized clinical prompts recommending DXA screening in men aged ≥ 70 years were implemented approximately a decade ago. This setting approximates routine screening rather than purely opportunistic referral.

We therefore aimed to evaluate the prevalence and skeletal distribution of low BMD in men aged ≥ 70 years within a large national health system, applying exclusion criteria to minimize secondary osteoporosis and reporting both site-specific and overall diagnostic classifications.

## Methods

### Study Design and Data Source

We conducted a retrospective cohort study of dual-energy X-ray absorptiometry (DXA) examinations performed between January 1, 2014 and December 31, 2024 at Assuta Medical Centers, using the MDClone© platform for structured data extraction. Within Maccabi Health Services, a computerized clinical recommendation supporting DXA screening in men aged ≥ 70 years was implemented approximately a decade ago. Most examinations for Maccabi members are performed at seven Assuta DXA facilities nationwide, providing broad geographic coverage and minimizing referral variability. In Israel, DXA testing is reimbursed through the national health basket for both men and women aged ≥ 60 years. This reimbursement structure facilitates access to bone mineral density (BMD) testing and reduces financial barriers to age-based evaluation.

### Study Population

The source population consisted of all men aged ≥ 70 years who underwent DXA assessment within Maccabi Health Services during the study period, as identified from the centralized electronic medical record database. To approximate primary age-based screening conditions and minimize inclusion of referral-driven or secondary osteoporosis evaluations, a sequential restriction process was applied.

First, the dataset was restricted to the first DXA examination performed at or after age 70 years for each individual that was labeled as a screening examination on the referral form, in order to avoid over-representation of follow-up studies. Second, examinations with structured or free-text documentation suggestive of secondary osteoporosis or non-screening indications were excluded. These included: (i) prior fracture, (ii) oncologic disease (solid or hematologic malignancy), (iii) chemotherapy or radiotherapy exposure, (iv) endocrine causes of secondary osteoporosis, including hypogonadism, hyperthyroidism, or hyperparathyroidism, (v) chronic systemic glucocorticoid therapy and (vi) chronic inflammatory conditions (e.g., rheumatoid arthritis, inflammatory bowel disease, steroid-treated pulmonary disease). Referral text and available clinical fields were examined to identify potential non-screening indications, including prior fracture evaluation, malignancy, glucocorticoid therapy, or other secondary osteoporosis conditions. Third, to evaluate the validity of the screening designation**,** a random sample of 200 DXA examinations labeled as screening was manually reviewed. This restriction strategy was designed to approximate an age-based screening population as closely as possible within real-world clinical data, while minimizing inclusion of referral-driven examinations or secondary osteoporosis evaluations. During the study period, approximately 50% of men aged ≥ 70 years within Maccabi Health Services underwent DXA assessment, supporting substantial uptake of screening recommendations and reinforcing the relevance of this cohort to real-world age-based screening conditions.

Finally, participants were required to have at least two valid skeletal site-groups available for analysis (lumbar spine, femoral neck, and/or total hip). Examinations lacking sufficient skeletal data were excluded, yielding a final analytic cohort of 29,906 men.

### BMD Measurement and Classification

Skeletal site-groups included the lumbar spine (L1–L4), the femoral neck (minimum T-score of left or right), and the total hip (minimum T-score of left or right). Bone mineral density was classified according to World Health Organization criteria. T-scores were categorized as normal (≥ − 1.0), osteopenia (− 1.0 to − 2.49), or osteoporosis (≤ − 2.5). T-scores were calculated using the manufacturer’s reference database based on the NHANES III female reference population, in accordance with widely adopted clinical practice and international recommendations. Prevalence estimates were reported for each skeletal site separately and according to the lowest T-score across all measured sites. The test date was defined as baseline.

### DXA Acquisition and Quality Control

All DXA examinations were performed at Assuta Medical Centers using GE Lunar densitometry systems (GE Healthcare, Madison, WI, USA). Standardized acquisition protocols were applied across sites. Quality assurance procedures included regular machine calibration in accordance with manufacturer guidelines and routine phantom scanning. All examinations were conducted and interpreted by an experienced team trained in bone densitometry, with certification aligned with International Society for Clinical Densitometry (ISCD) standards. Cross-site consistency was maintained through standardized protocols and centralized oversight.

### Statistical Analysis

All analyses were descriptive. Continuous variables are presented as mean ± standard deviation (SD), and categorical variables as counts and proportions. Osteoporosis prevalence was calculated overall and stratified by age group (70–74, 75–79, and ≥ 80 years). Prevalence estimates are presented with 95% confidence intervals. Analyses were performed using IBM SPSS Statistics for Windows, version 27 (IBM Corp., Armonk, NY, USA).

## Results

### Study Population and Cohort Flow

The study population consisted of 30,352 men aged ≥ 70 years who underwent a first DXA examination labeled as a screening assessment between 2014 and 2024 within Maccabi Health Services. This cohort already excluded individuals with known oncologic disease, prior fractures, inflammatory conditions, or other predefined clinical indications for DXA based on structured electronic records. To further approximate primary age-based screening conditions, additional screening-enrichment filtering was applied using referral free-text review and diagnostic fields in order to identify potential secondary causes of osteoporosis or non-screening indications. After this refinement process, 29,906 individuals remained eligible for analysis. All 200 manually reviewed examinations (100%) were confirmed to represent age-based screening referrals, with no evidence of fracture evaluation, malignancy, or other secondary osteoporosis indications.

### Population Characteristics

The mean age at the time of DXA assessment was 74.2 ± 4.5 years. Age distribution demonstrated that 62.8% of participants were aged 70–74 years, 22.2% were aged 75–79 years, and 15.0% were aged ≥ 80 years. BMI data were available for a subset of participants (n = 12,352), with a mean BMI of 27.3 ± 3.9 kg/m^2^. Background disease information was available for 16,663 participants (54.9%). Diabetes mellitus was documented in 3,069 individuals (10.1%), hypertension in 7,021 (23.1%) and hyperlipidemia in 3,694 individuals (12.2%). Smoking status was documented in 2600 participants. Baseline demographic and clinical characteristics of the cohort are summarized in Table 1.

**Table 1:** Baseline Characteristics of the study population (n = 29,906) of men aged ≥ 70 years with ≥ 2 valid skeletal site-groups

Characteristic	Value
Number of participants	29,906
Age, years (mean ± SD)	74.2 ± 4.5
Age group, n (%)	
70–74 years	18,781 (62.8%)
75–79 years	6,639 (22.2%)
≥ 80 years	4,486 (15%)
Body mass index, kg/m^2^ (mean ± SD)	27.3 ± 3.9
Diabetes mellitus, n (%)	3,069 (10.1%)
Hypertension, n (%)	7,021 (23.1%)
Hyperlipidemia, n (%)	3,694 (12.2%)
Smoking status, n (%)	
Non-smoker	2,267 (87.2%)
Current smoker	213 (8.2%)
Former smoker	47 (1.8%)

Background disease information was available for 16,663 participants (54.9%).*

### Overall and Age-Stratified Prevalence of Osteoporosis and Osteopenia

Using the lowest T-score across the lumbar spine, femoral neck, and total hip, osteoporosis was identified in 4,940 men, corresponding to an overall prevalence of 16.5% (95% CI 16.1–16.9%).

Osteopenia was identified in 15,353 men, corresponding to a prevalence of 51.3% (95% CI 50.7–51.9%). Overall, low bone mass (osteopenia or osteoporosis) was identified in 67.9% of men aged ≥ 70 years in the screening-enriched cohort.

**Fig. 1:**
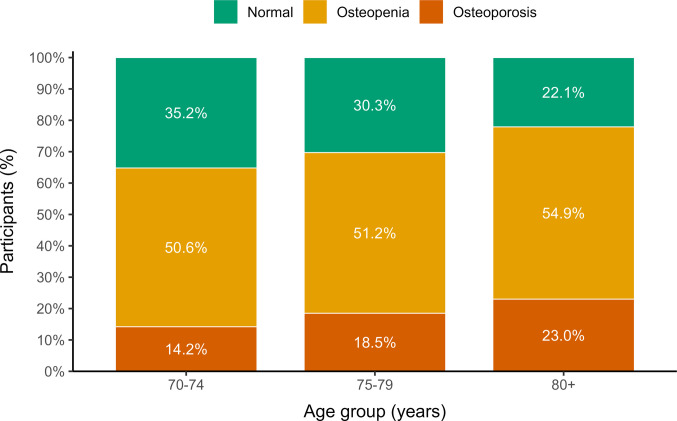
Age-stratified prevalence of osteoporosis and osteopenia in men aged ≥ 70 years

Age-stratified analysis demonstrated a progressive deterioration in bone mineral density with advancing age. While osteoporosis prevalence increased markedly with age, osteopenia remained highly prevalent across all age groups, indicating a substantial burden of low bone mass even among younger elderly men. The age gradient of osteoporosis prevalence is illustrated in Fig. 1.

While osteoporosis prevalence increased markedly with advancing age, osteopenia remained highly prevalent across all age groups, indicating that a substantial proportion of older men already exhibit reduced bone mineral density before reaching the diagnostic threshold for osteoporosis.

### Site-Specific Distribution of Bone Mineral Density

**Fig. 2:**
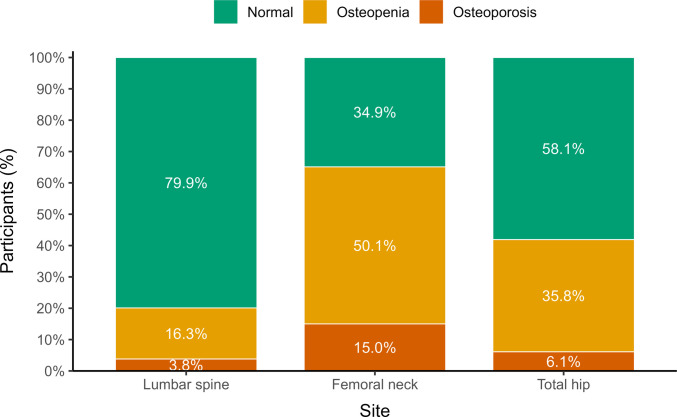
Bone mineral density classification by skeletal site

Site-specific analysis demonstrated substantial variation in bone mineral density classification across skeletal regions. At the lumbar spine (n = 29,581), 79.9% of men had normal BMD, 16.3% had osteopenia, and 3.8% met criteria for osteoporosis. At the femoral neck (n = 29,906), 34.9% were classified as normal, 50.1% had osteopenia, and 15.0% had osteoporosis. At the total hip (n = 29,903), 58.1% were normal, 35.8% had osteopenia, and 6.1% had osteoporosis (Fig. 2.) Overall, the femoral neck demonstrated the highest prevalence of both osteopenia and osteoporosis, whereas the lumbar spine showed the lowest prevalence of osteoporosis.

## Discussion

In this large cohort of men aged ≥ 70 years undergoing DXA evaluation within a national healthcare system, osteoporosis was identified in 16.5% of participants using the lowest T-score across measured skeletal sites, while approximately half of the population demonstrated osteopenia. Together, these findings indicate that nearly two-thirds of older men exhibited reduced bone mineral density. A pronounced age gradient was observed, with femoral neck osteoporosis increasing from 12.4% in men aged 70–74 years to 26.1% among those aged ≥ 80 years. This pattern parallels epidemiologic data demonstrating sharply rising hip fracture incidence with advancing age in men. The predominance of femoral neck involvement is clinically important given the well-documented excess mortality, functional decline, and long-term disability associated with hip fractures in men (2, 14, 15).

Comparative data from large population-based cohorts, including the MrOS study (9, 10) and analyses from the U.S. NHANES (11, 12), have reported osteoporosis prevalence in older men generally ranging from approximately 10–20%, depending on age, skeletal site, and diagnostic definition. The prevalence observed in our study falls within the upper range of these estimates and likely reflects the structured availability of DXA within a healthcare system incorporating computerized clinical recommendations supporting bone density evaluation in men aged ≥ 70 years, thereby supporting the external validity of the present results. Notably, the prevalence of osteoporosis observed in men aged ≥ 70 years in our cohort (16.5%) is comparable to that reported in women aged ≥ 50 years in large population-based studies, including NHANES data demonstrating osteoporosis prevalence of approximately 19–20% and low bone mass in approximately half of postmenopausal women (11, 12, 16).

Although randomized screening trials in men remain limited, disease prevalence represents a fundamental component of screening evaluation. The magnitude and age distribution of osteoporosis observed in this cohort provide important real-world epidemiologic context for ongoing discussions regarding skeletal risk assessment in older men. Notably, the high prevalence of osteopenia across all age groups suggests that a large proportion of older men already exhibit reduced bone mass prior to reaching the diagnostic threshold for osteoporosis, highlighting a potential window for earlier fracture prevention strategies. Identification of these individuals may provide an opportunity for earlier risk stratification and targeted fracture prevention strategies, particularly when combined with clinical risk assessment tools such as FRAX (17). Importantly, these findings provide epidemiologic context relevant to the current uncertainty surrounding osteoporosis screening in men highlighted by the recent USPSTF recommendation, which cited insufficient evidence despite recognition of substantial disease burden.

The lower prevalence observed at the lumbar spine compared with the femoral neck likely reflects age-related degenerative changes that may artifactually elevate lumbar spine bone mineral density measurements, potentially masking underlying skeletal fragility. This phenomenon has been widely described in aging populations and reinforces the importance of hip-site assessment when evaluating osteoporosis risk in older adults.

This study has several important strengths. It includes one of the largest screening-enriched cohorts of men aged ≥ 70 years undergoing DXA assessment, providing robust estimates of bone mineral density distribution in this population. The use of a centralized electronic medical record database enabled systematic filtering of potential secondary osteoporosis causes through both structured variables and referral free-text review, allowing construction of a cohort that closely approximates primary age-based screening conditions. In addition, standardized imaging protocols across a national network of facilities improved measurement consistency.

Several limitations should be acknowledged. The retrospective cross-sectional design precludes assessment of longitudinal fracture outcomes, which would further clarify the clinical implications of osteoporosis detection in this population. Although several restrictions were applied to approximate primary screening conditions, the study population may not represent a formal population-based screening program, and some degree of referral-related selection may persist.

## Conclusion

Among men aged ≥ 70 years, low bone mineral density is highly prevalent, with osteoporosis affecting approximately one in six individuals and osteopenia affecting about half of the population. Osteoporosis prevalence increases markedly with advancing age and is most pronounced at the femoral neck. These large-scale real-world data provide important epidemiologic evidence regarding the burden and skeletal distribution of osteoporosis in older men and contribute to ongoing discussions regarding optimal strategies for skeletal risk assessment in this population. The presence of substantial bone loss already at age 70 suggests a potential window of opportunity for early detection and preventive intervention, before the sharp increase in fracture risk observed in the subsequent decades of life.

## AI Disclosure Statement

During the preparation of this manuscript, generative artificial intelligence tools (ChatGPT 5.2) were used to assist with language refinement and structural editing. All content was reviewed and validated by the authors, who take full responsibility for the integrity and accuracy of the work.

## Ethics

This study was conducted in accordance with the Declaration of Helsinki. The study protocol was approved by the institutional review board (IRB) of Assuta Medical Centers and the relevant ethics committee of Maccabi Health Services. Due to the retrospective nature of the study and use of de-identified registry data, the requirement for informed consent was waived by the approving ethics committee.

## Conflict of Interest

V.R., Z.V., M.S., Y.G., A.M., G.C., and I.G. declare that they have no conflicts of interest.

## Data Availability

The data that support the findings of this study are derived from Maccabi Health Services and Assuta Medical Centers and are not publicly available due to institutional and privacy regulations. De-identified data may be made available from the corresponding author upon reasonable request and subject to institutional approval.
